# Micro-nanoplastics in Cardiovascular Disease: A Critical Update from Environmental Exposure to Clinical Implications

**DOI:** 10.1007/s12012-026-10149-0

**Published:** 2026-06-16

**Authors:** Zeeba Saeed, Marco Gatta, Annalisa Bruno, Melania Dovizio, Cristina Milillo, Amedeo Amedei, Giulia Renda, Patrizia Ballerini

**Affiliations:** 1 Center for Advanced Studies and Technology (CAST), “G. d’Annunzio” University of Chieti-Pescara, Chieti, Italy; 2 Department of Innovative Technologies in Medicine & Dentistry, “G. d’Annunzio” University of Chieti-Pescara, Chieti, Italy; 3 Department of Experimental and Clinical Medicine, University of Florence, Florence, Italy; 4 Department of Neurosciences, Imaging and Clinical Sciences, “G. d’Annunzio” University of Chieti-Pescara, Via dei Vestini, 31, Chieti, 66100 Italy

**Keywords:** Micro-nanoplastic, Cardiovascular disease, Inflammation, Endothelial dysfunction, Platelet, Coagulation cascade

## Abstract

Micro- and nanoplastics (MNPs) are emerging environmental contaminants that may contribute to cardiovascular risk. Observational human studies have reported the presence of MNPs in patients with myocardial infarctions, acute coronary syndrome, coronary artery disease, extracranial artery stenosis, and asymptomatic extracranial internal carotid artery stenosis, where higher MNP levels have been associated with inflammatory and coagulation-related biomarkers and disease severity. Moreover, experimental in vivo and in vitro studies provide biological plausibility for these associations by showing that MNP exposure may promote endothelial dysfunction, oxidative stress, inflammation, platelet activation, coagulation abnormalities, cardiometabolic alterations, myocardial remodeling, fibrosis, and hypertension. However, major uncertainties remain regarding environmentally relevant exposure levels, long-term human effects, and the translational relevance of current experimental findings. This review critically summarizes the current evidence on the potential cardiovascular effects of MNPs, focusing mainly on endothelial dysfunction, platelet activation, the coagulation cascade, inflammation, gut dysbiosis, and alterations in lipid metabolism. We also discuss emerging therapeutic perspectives, iatrogenic exposure through cardiovascular medical devices, and the major methodological and translational limitations that currently hinder definitive cardiovascular risk assessment in humans.

## Introduction

Micro- and nano-plastics (MNPs) are widespread environmental pollutants that have garnered increasing scientific interest because of emerging experimental and epidemiological evidence suggesting potential adverse effects on human health [1]. A broad range of plastic polymers, including polythene, polypropylene (PP), polyethylene terephthalate (PET), high-density polyethylene (PE), low-density PE, polyvinyl chloride (PVC), acrylic, and polystyrene (PS), is employed across industrial, medical, and consumer applications [2]. MNPs are intentionally manufactured particles used in more industrial and consumer applications. Engineered for resilience, plastics are resilient to natural degradation processes. Nonetheless, they gradually fragment through physical and chemical mechanisms into microplastics (MPs; <5 mm) and nanoplastics (NPs; < 1 μm) [3, 4]. MNPs are widespread in the air, water, and food [5–7].

MNPs enter the human body through different routes, i.e., ingestion, inhalation, and skin exposure, and then interact with tissues and organs [8]. However, a current study reported that a range of medical practices, such as percutaneous coronary intervention (PCI), could be a new MP exposure pathway in humans. Unlike other routes of exposure, PCI may provide a direct intravascular pathway for MNPs, bypassing physiological epithelial barriers. Preliminary evidence suggests that PCI procedures may introduce substantial numbers of MPs into the bloodstream [9]. It can also be inferred that other interventional treatments may allow MPs to be exposed to the body, given the similarity in device materials and their procedures [9]. The MNP presence has been reported in several human tissues, including intestine, placenta, lungs, kidney, brain, and liver, as well as in breast milk, saliva, urine, blood, umbilical cord blood, seminal fluid, amniotic fluid, and thrombus samples [1, 10–16]. The detection of MNPs in multiple organs and tissues has raised concerns about their potential contributions to chronic diseases, including cardiovascular diseases (CVD), respiratory diseases, immune system disorders, chronic inflammation, effects on endocrine glands leading to hormonal imbalances, and metabolic dysfunctions [17–20]. The size and physicochemical properties of the MNPs affect their ability to penetrate multiple tissues and organs [21, 22]. Plastics with a diameter ≤ 100 nm can cross biological barriers, enter the bloodstream, and induce more pronounced biological effects than larger particles [23]. For example, NPs may preferentially accumulate at atherosclerotic sites compared to MPs [11]. This finding, along with the MP detection in aortic thrombi [24], further supports a potential association between MNPs and CVD [1]. The presence of PS-MPs in carotid artery tissues is associated with a higher risk of heart attacks, strokes, or even higher mortality rates [11]. Experimental data from preclinical models indicate that MNPs induce oxidative stress, platelet aggregation, cell senescence, and inflammatory responses in endothelial and immune cells, leading to a range of cardiovascular and metabolic alterations that can cause disease and premature death [25].

MNPs negatively affect human blood by denaturing plasma proteins, causing hemolysis, decreasing immunity, impairing blood coagulation, promoting thrombosis, and damaging the vascular endothelium, which, in turn, result in life-threatening diseases [26]. When NPs enter a biological fluid, e.g., plasma, different biomolecules can adhere to them, making the “protein corona” which might be biologically more active than the NP itself [27]. The NP size and curvature play a key role in affecting the composition of bound protein in the corona [28]. The protein coronas surrounding different PS-NPs in plasma have been identified, comprising proteins associated with lipid metabolism, the complement system, and blood coagulation, among others [27]. The analysis of MPs isolated from circulation by Sodium dodecyl sulfate polyacrylamide gel electrophoresis revealed that the corona is mostly composed of fibrinogen, globulins, and albumin [29]. A hydrophilic protein corona may mitigate the immune response and extend the circulation time of MNPs in the bloodstream, with consequent effects on their biodistribution and potential toxicity [30].

NPs can interact directly or indirectly with circulating blood elements, including platelets [31], leukocytes, erythrocytes, complement proteins, and immunoglobulins, altering their structure and function [32]. Additionally, NPs bind to red blood cells (RBCs), causing hemolysis by altering erythrocyte structure, resulting in numerous aberrant shapes [33]. The NP interaction with coagulation factors induces pro-coagulant to anti-coagulant effects, depending on protein sorption onto the particle surface and the size and surface chemistry of the NPs [31, 34]. The different functional groups on the PS-NP surface induce RBC hemolysis with diverse potencies: the amine group was found to be more potent at altering RBCs; however, the mechanism underlying the amine-modified PS-NPs’ higher potency remains unknown. Interestingly, 50 nm amine-modified PS-NPs were less effective than 100 nm PS-NPs in causing RBC alterations, suggesting that surface modification, size, and surface area may be critical determinants of particle-induced toxicity. Future research is warranted to further explore the effects of various plastic shapes on RBCs and thrombotic diseases [35].

NPs can cross the endothelial barrier either via active trans-endothelial transport or passively through inter-endothelial gaps. These gaps can reach up to 2000 nm, allowing for protein translocation [36].

MPs are well established in animal models for inducing oxidative stress in numerous organs and promoting inflammation. However, the mechanisms by which they affect human health, especially through changes in thrombosis and hemostasis in vivo, remain poorly investigated. Further research is required to clarify the association between MPs and human health, with a focus on their impact on the coagulation system [37, 38].

This systematic review critically examines the available clinical and preclinical evidence on the potential cardiovascular effects of MNP exposure. We focus on their involvement in endothelial dysfunction, thrombosis, inflammatory responses, alterations in lipid metabolism, and hemostatic abnormalities, while also highlighting the limitations and translational challenges of the currently available evidence. The insights provided on this emerging topic are intended to inform and inspire future research efforts to better clarify the mechanisms underlying MNPs-related cardiovascular risk.

## Methods

**Fig. 1:**
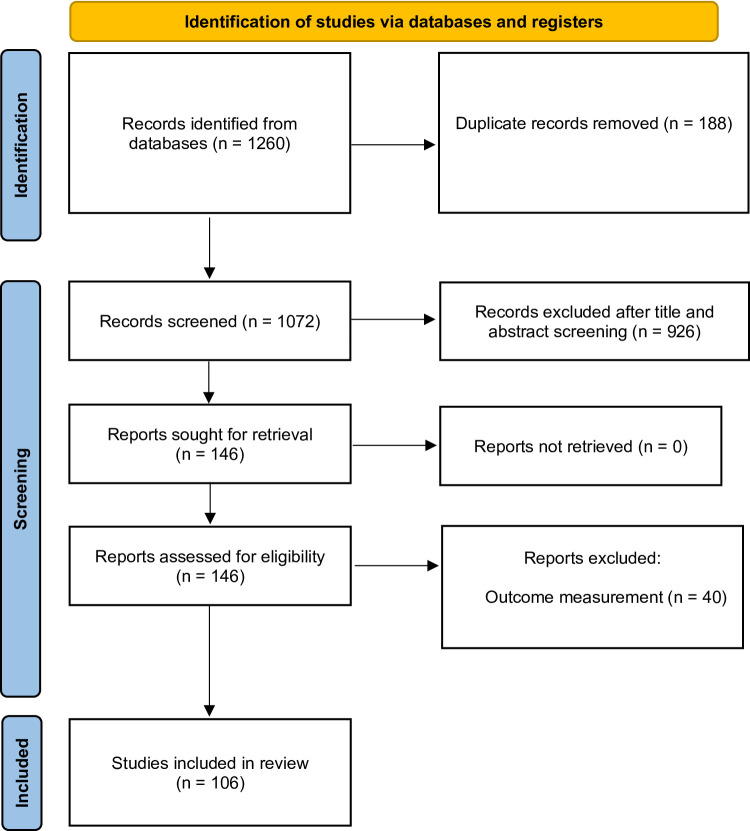
PRISMA 2020 flow diagram of study selection process

This review synthesized current evidence on the cardiovascular toxicity of MNPs. A comprehensive literature search was carried out from February 2025 to February 2026 using mainly PubMed, ScienceDirect, and Google Scholar. The total number of publications identified from the literature search was 1260. 146 papers were retrieved for full-text review, and 106 papers were included in the review (Fig. 1).

The search strategy focused on selecting the studies that particularly addressed the following outcomes: (a) MNPs induced CVD in human and animal models, (b) MNPs triggered thrombotic and hemorrhagic complications, coagulation abnormalities, platelets, and endothelial dysfunction, (c) MNPs associated with dysregulated gut-heart axis and altered metabolism causing CVD, (d) Therapeutic targets for MNPs induced CVD, (e) MNPs exposure via medical devices in cardiovascular procedures.

The results were presented systematically. The search terms used were ‘cardiovascular disease’, ‘thrombosis’, ‘hemorrhage’, ‘blood cells’, ‘platelets’, ‘endothelial dysfunction’, ‘coagulation cascade’, ‘hypertension’, and ‘medical device’ in combination with the term ‘microplastic’ or ‘nanoplastic’.

### Cardiovascular Toxicity of MNPs

**Fig. 2:**
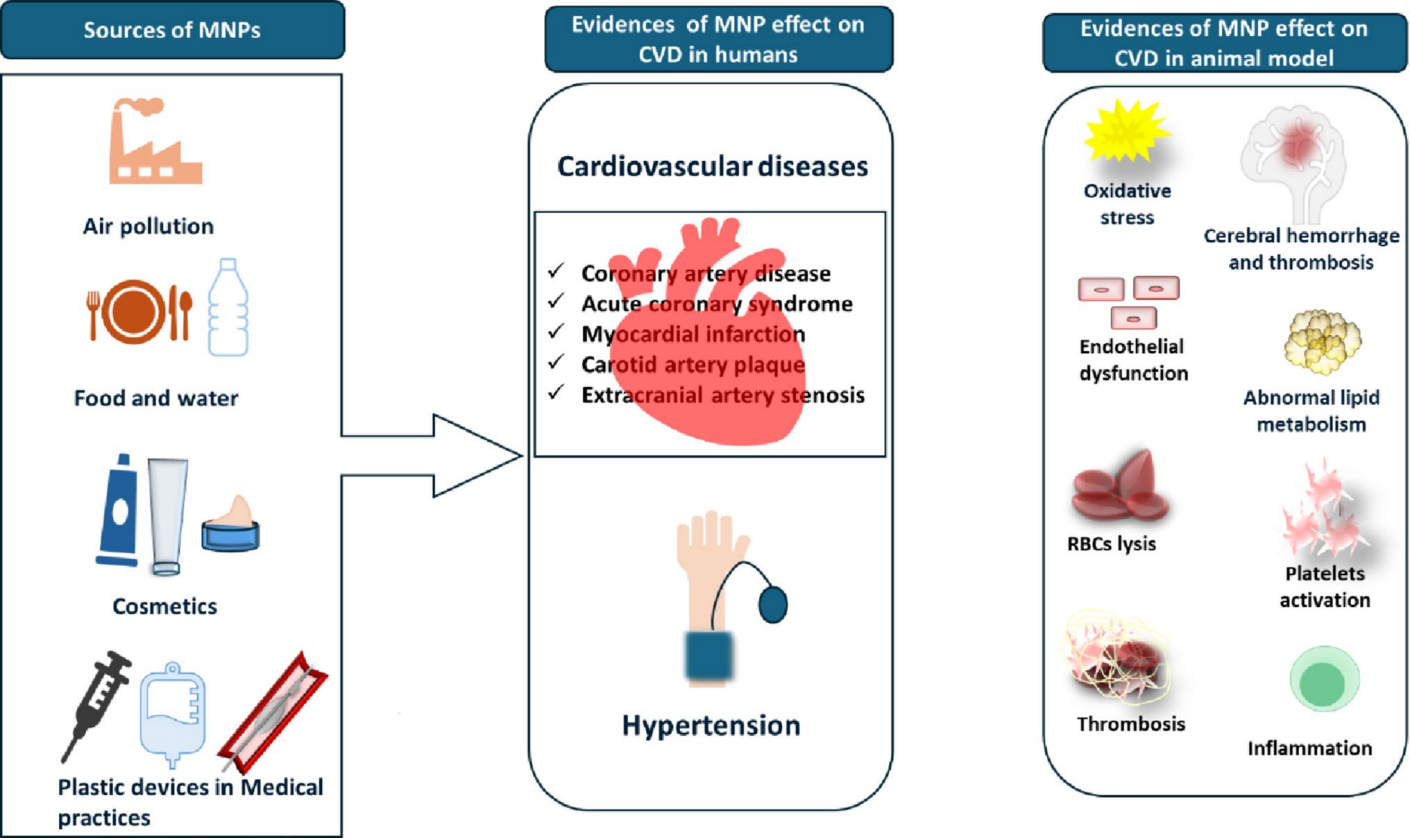
Schematic illustration of the effects of micro-nanoplastics (MNPs) on cardiovascular complications in human and animal models. Several kinds of plastic are utilized in industrial, medical, and consumer goods. MNPs are widespread in the air, water, food, and drinking water. Current observational clinical studies have shown that the presence of MPs in carotid artery plaques of patients predisposes them to a higher risk of cardiovascular events. MNPs trigger cardiovascular toxicity, which leads to the development of cardiac dysfunction, myocardial fibrosis, vascular thrombosis, and vessel damage

Experimental and emerging clinical evidence suggest that MNP exposure may contribute to cardiovascular toxicity, including cardiac dysfunction, myocardial fibrosis, vascular stenosis, thrombosis, and endothelial injury (Fig. 2) [39]. Preclinical studies indicate that MNPs can impair vascular growth and repair by generating reactive oxygen species (ROS), triggering inflammatory responses, and inducing pyroptosis [40]. A recent study documented that US coastline counties with higher concentrations of marine MPs showed an association with higher prevalence of type 2 diabetes, coronary artery disease, and stroke than those with lower MP concentrations [41]. Current observational clinical studies have shown that the presence of MP in carotid artery plaques increases patients’ risk of cardiovascular events [11]. The presence of PE and PVC MNPs in the atheroma of patients with asymptomatic high-grade (> 70%) carotid artery stenosis was associated with a higher risk for a primary end-point (non-fatal MI, non-fatal stroke, or death from any cause) event compared to those patients having no MNPs within the plaque (20.0% vs. 7.5%) [11]. Supporting these observations, Yu et al. [42] demonstrated significantly elevated blood levels of PVC and polyamide 66 (PA66) in patients with extracranial artery stenosis (ECAS). The significant increase in D-dimer levels and prolonged thrombin time in the ECAS group compared to the control group may indicate the ongoing thrombotic activity and endothelial dysfunction, such as atherosclerosis. Moreover, the strong correlation between MPs’ blood concentrations and ECAS severity suggests a possible role of MPs in the pathophysiology of ECAS, but further research is warranted to clarify the mechanisms by which MPs contribute to the deterioration of vascular health. Understanding the interactions between MPs and molecular/cellular pathways in atherosclerosis will be critical [42].

Moreover, patients with acute coronary syndrome (ACS) showed increased concentrations of MPs compared to controls. Patients with intermediate or high risk of coronary artery disease had significantly increased MP accumulations compared to their low-risk counterparts. Additionally, increased MP levels were significantly correlated with elevated inflammatory factors. These findings support a possible association between MP burden, vascular pathology complexity, and immunoinflammatory activation in ACS patients. However, further research is needed to understand the mechanism behind this association [43].

Patients with myocardial infarction (MI) showed the presence of MP in the coronary blood. PVC, PE, PA 66, and PS were more prevalent among patients. PVC levels were positively associated with proinflammatory markers and monocyte/macrophage infiltration. They were also associated with a higher odd of MACE (OR: 1.090, 95% CI: 1.032–1.152, *P =* 0.002). For every 10-unit increase in PVC concentration, the MACE risk increases by 1.374-fold (95% CI: 1.366–4.128). These findings suggest that PVC exposure may contribute to adverse cardiovascular outcomes in MI patients [22]. Further research is warranted to determine whether PVC preferentially accumulates in coronary arteries and to compare its pro-inflammatory properties with those of other MNPs. The right mechanism by which MNPs might worsen the prognosis of MI patients is not clearly known [22].

Table 1 shows the detected concentrations of different MNPs in healthy and CVD patients. Similarly, Gu et al. [1] indicated the presence of PA66, PET, PVC, and PS as major MPs in the coronary blood of MI patients. Their study on PET-MNPs in MI showed that PET-NPs induce the secretion of inflammatory factors by peripheral monocytes. PET-NPs also inhibited AC16 cell proliferation and promoted hypoxia-induced AC16 cell apoptosis.

**Table 1:** Concentrations of different micro-nanoplastics (MNPs) in healthy and cardiovascular disease patients

Study design/population characteristics	Samples type	Technique	Total plastics	PVC	PA	PA66	PE	PS	PET
**Observational Clinical Study** Extracranial artery stenosis patients (*n* = 20)	Arterial blood	Py-GC/MS/LDIR	174.89 ± 24.95 μg/g	134.79 ± 23.25 μg/g		40.03 ± 7.31 μg/g			
Control (*n* = 10)			79.82 ± 31.73 μg/g	60.80 ± 19.83 μg/g		19.03 ± 13.54 μg/g			
**Prospective**,** multicenter**,** observational study** Asymptomatic extracranial high-grade (> 70%) internal carotid artery stenosis (*n* = 257; detected in 150)	Carotid plaque	Py-GC/MS/TEM		5.2 ± 2.4 μg/mg of plaque			21.7 ± 24.5 μg/mg of plaque		
**Prospective**,** observational study** Patients with myocardial infarction (*n* = 110)	Coronary blood	Py-GC/MS		10.1 ± 7.7 mg/kg		20.89 ± 12.87 mg/kg	45.61 ± 26.25 mg/kg	2.47 ± 6.38 mg/kg	
	Coronary thrombi			15.67 ± 13.26 mg/kg		32.28 ± 14.57 mg/kg	18.33 ± 10.13 mg/kg	1.26 ± 0.67 mg/kg	
**Experimental study** Patients with ST-segment elevation myocardial infarction (*n* = 34; MPs analyzed in 10 patients) Healthy donors as controls for in vitro experiments (*n* = 34)	Coronary blood	Py-GC/MS		21.83 ± 1.68–36.04 ± 1.31 mg/kg		6.03 ± 0.99–90.07 ± 2.97 mg/kg		1.33 ± 0.10–8.67 ± 0.71 mg/kg	1.38 ± 0.2–12.38 ± 1.1 mg/kg
**Experimental study** Healthy population (*n* = 36; MPs detected in 32)	Blood	µ-FTIR/μ-Raman	4.2 N/1 ml		0.1 N/mL		0.7 N/mL	1.7 N/mL	0.3 N/mL
**Experimental Study** Patients with acute chest pain (*n* = 101)	Preoperative venous blood samples	Py-GC/MS	150.08 μg/g of blood	28.50%		0.9%	58.51%	5.93%	
Controls (based on angiography no coronary artery disease (*n* = 19)			100.13 μg/g of blood	18.13%		0.82%	74.40%	2.04%	
Aute coronary syndromes (*n* = 82)			161.65 μg/g	29.98%		0.94%	56.23%	6.49%	
**Experimental Study** Coronary artery disease undergoing for the first time PCI (*n* = 23) Pre-PCI blood samples	blood from peripheral vessel	Laser direct infrared (LDIR)	4.96 ± 3.40 N/10 mL of blood		1.22 ± 0.95 N/10 mL of blood		0.43 ± 0.51 N/10 mL of blood		1.04 ± 1.11 N/10 mL of blood
Post-PCI blood samples			93.57 ± 35.95 N/10 mL of blood		63.52 ± 28.06 N/10 mL of blood		11.61 ± 5.61 N/10 mL of blood		4.96 ± 3.55 N/10 mL of blood

Study design/population characteristics	PP	PU	Particle size (µm)	Exposure route	Endpoint	Limitation	Citation
**Observational Clinical Study** Extracranial artery stenosis patients (*n* = 20)			20–100	Environmental or occupational exposure	Strong correlation between the levels of MPs in the blood and the severity of ECAS	Small sample size and single region Py-GC/MS, is a destructive testing method LDIR cannot detect MPs smaller than 20 μm.	Yu et al. [42]
Control (*n* = 10)					ECAS group had a higher level of D- and longer thrombin time		
**Prospective**,** multicenter**,** observational study** Asymptomatic extracranial high-grade (> 70%) internal carotid artery stenosis (*n* = 257; detected in 150)			< 1	Not given	MNPs showed an association with the primary endpoint (nonfatal MI, nonfatal stroke, or death from any cause) Levels of PE showed correlation with secondary end point IL-18, IL-1β, IL-6, TNF-α, CD68, CD3, and collagen	Residual risk of contamination might exist Lack of socioeconomic data These findings may not represent general population Did not explore source of MNPs.	Marfella et al. [11]
**Prospective**,** observational study** Patients with myocardial infarction (*n* = 110)			Not given	Not given	MACE in MI patients, including recurrent MI, heart failure, reduced cardiac function, and non-accidental death showed significant association with PVC MNPs PVC MNPs levels were positively correlated with proinflammatory cytokines IL-1β, IL-6, IL-18, and TNF-α, in coronary blood and CD3 and CD68 in coronary thrombi	Small sample size and single-center design Possibility of laboratory contamination Did not explore the potential sources of MNPs Inability to establish a cause-and-effect relationship between MNPs concentration and the incidence of MACE in patients with MI Did not assessed the precise mechanisms by which MNPs might worsen the prognosis of these patients.	Zhang et al. [22]
**Experimental study** Patients with ST-segment elevation myocardial infarction (*n* = 34; MPs analyzed in 10 patients) Healthy donors as controls for in vitro experiments (*n* = 34)			Not given	Not given	In vitro experiments showed that PET NPs can stimulate human PBMCs, resulting in immune cell imbalance in MI patients and leading to immune disorders and ventricular remodelling	Did not explore the exposure route of MPs to the coronary circulatory system.	Gu et al. [1]
**Experimental study** Healthy population (*n* = 36; MPs detected in 32)	1.4 N/mL		10– ≥ 100	Use of plastic food containers	Higher MPs level was associated with altered coagulability Increased MPs burden in the blood was significantly correlated with increased aPTT, C-reactive protein, and fibrinogen	Study samples were not generalizable and were conveniently recruited Laboratory contamination cannot be completely excluded μ-FTIR method, cannot detect MPs smaller than 5–20 μm.	Lee et al. [106]
**Experimental Study** Patients with acute chest pain (*n* = 101)	6.14%		Not given	Not given	MPs are associated with complexity of vascular pathologies and immunoinflammatory status in ACS patients MPs are significantly corelated with enhanced IL-6 and IL-12p70 contents, alongside elevated B lymphocyte and natural killer cell counts.	Blood samples collected at admission were analyzed for MP content detection to minimize potential contamination of MP from medical procedure exposure This approach limited the analysis to explore the correlation between blood MP levels and ACS at different time point Due to invasive nature of coronary angiography the inclusion of healthy participants in this study was constrained, which restricted the sample size of the control group and failed to clarify the potential risks of MPs to healthy populations Did not investigate the effects of MPs on human macrophage phenotypes.	Yang et al. [43]
Controls (based on angiography no coronary artery disease (*n* = 19)	4.61%						
Aute coronary syndromes (*n* = 82)	6.37%						
**Experimental Study** Coronary artery disease undergoing for the first time PCI (*n* = 23) Pre-PCI blood samples		0.61 ± 1.20 N/10 mL of blood	50	PCI	The levels of MPs in the blood were significantly elevated post-PCI compared to pre-PCI.	68% nitric acid used to disintegrate the cells and proteins in the blood which may alter the surface of the MPs causing changes in the infrared spectra and may cause the MPs to fragment, leading to an underestimation of the concentration of the MPs LDIR method cannot detect MPs particles smaller than 20 μm Each device selected for the study was from a single manufacturer.	Liu et al. [9]
Post-PCI blood samples		10.70 ± 4.58 N/10 mL of blood	213				

In addition to observational human studies, several experimental animal models have provided mechanistic insights into the toxicity of MNPs in CVD.

PET-NP exposure resulted in a higher mortality incidence in both MI + NP-exposed mice, who received 50 μg/ml oral PET-NPs, and in mice exposed to PET-NPs by aerosol inhalation (12-h continuous exposure to PET-NPs at 1 mg/m3 by aerosol inhalation every day for 3 months). PET-NPs activated the NLRP3 inflammasome and exacerbated post-MI inflammation and fibrosis. Macrogenetic sequencing and metabolomics analyses revealed reprogramming of the intestinal and lung microbiota and metabolome in MI mice, leading to chronic inflammation. Rats exposed to different concentrations (0.5, 5, and 50 mg/kg/d) of PS-MPs for 90 days showed no significant pathological alterations in myocardial cells or blood vessels. However, the high-dose PS-MPs group (50 mg/kg/d) showed a significant increase in creatine phosphokinase isoenzyme activity. No significant increase in cardiac troponin T levels was observed. High-dose accumulation over a longer period might lead to myocardial pathological alterations due to impaired cellular function [44]. In agreement with these results, a recent study documented that PET-NP ingestion was also associated with alterations in the lung microbiome, including increased *Pseudomonas* and changes in microbial energy metabolism and nitrogen utilization [45].

Wang et al. [46] found dose-dependent vascular wall thickening, disordered cell arrangement, and inflammatory cell infiltration in mice across the PS, amine-modified PS, and carboxylated-modified PS-MP groups. These findings suggest that surface-modified MPs may contribute to vascular injury by disrupting the endothelial barrier and eliciting inflammatory responses.

Exposure of 0.1 μm airborne PS-NPs (28.4 ± 1.4 mg/m^3^) to Improved Castle Road (ICR) male mice for 1 and 2 weeks resulted in decreased heart mass compared to control. However, the heart rate significantly diminished in the 2-week group compared to the control group [413 ± 24 beats per minute (bpm) vs. 517 ± 15 bpm]. The histologic examination unveiled signs of ventricular hypertrophy, ventricular myocardial hypertrophy, and myocardial necrotic fibrosis, leading to heart failure. Mechanistic studies suggested that extracellular matrix (ECM) receptor interactions and the PI3K/AKT/BCL-2 signaling pathway are involved in NPs-induced cardiac dysfunction [47]. Table 2 summarizes animal model studies investigating MNP-induced toxicity.

**Table 2:** Summary of effects of micro-nano plastics (MNPs) toxicity in animal in vivo studies

Models	MNPs size	MNPs type	Concentrations	Exposure method	Exposure time	Condition studied	Key findings	Underlying mechanism	Limitation	Citation
Improved Castle Road (ICR) male mice	0.1 μm	PS	28.4 ± 1.4 mg/m^3^ (daily inhalation 1 mg/day)	Exposure chamber	1 OR 2 weeks	Heart failure	Heart mass and heart rate reduced Conduction block Ventricular and myocardial hypertrophy Myocardial necrotic fibrosis Increases in cardiac ejection fraction and stroke volume.	PI3K/AKT/BCL-2 pathway	Not given	Chang et al. [47]
Male BALB/c mice	80 nm	PS	5, 10 and 20 mg/kg body weight	Caudal vein injection	14 days	Vascular injury and prethrombotic state	Reduced Body weight Inflammatory damage Vascular injury Destruction of endothelial barrier Coagulation dysfunction PS-NH_2_ has a significant biological toxicity	Activation of JAK1/STAT3/TF signaling pathway	Limited to PS-NPs Dose of toxic effects of NPs is relatively high Short time exposure	Wang et al. [46]
		PS-NH_2_	0.05, 0.5 and 5 mg/kg body weight							
		PS-COOH	0.05, 0.5 and 5 mg/kg body weight							
Female SD rats	0.3 μm	PS	0.01 mg/g body weight/day	Gavage	42 days	Blood pressure and myocardial hypertrophy	Elevated blood pressure Myocardial hypertrophy Increased oxidative damage to the heart Decrease in cardiac BK, eNOS, and serum NO levels PS-NH_2_ caused more significant cardiotoxic effects.	Down-regulation of BK-NO pathway and activation of MAPK-ERK	Not given	Du et al. [51]
		PS-NH_2_								
		PS-COOH								
BALB/c mice	5 μm	PS	1 mg/L and 2 mg/L	Drinking water	8 weeks	Hypertension and cardiac remodeling	Systolic, diastolic, and mean blood pressure elevation Cardiac hypertrophy, vascular remodeling, and myocardial fibrosis Dysbiosis in gut microbiota Higher dose of MPs showed adverse effects.		Not given	Wang et al. [50]
1-day-old chicks (half male and female)	5 μm	PS	1, 10 and 100 mg/L	In drinking water	42 days	Cerebral hemorrhage	Intracerebral hemorrhage Microthrombi formation Loss of Purkinje cells Inflammation Mitochnondrial dysfunction Pyroptosis High PS concentration exhibited more severe effect.	Activation of ASC-NLRP3-GSDMD signaling pathway and activation of AMPK signaling pathway.	Not given	Yin et al. [64]
Male wild-type C57BL/6J mice	5 μm, 2 μm, 80 nm	PS	(2, 0.5 and 0.1 mg/mL) (50, 25 and 5 μg/mL)	Gavage and intravenous injection		Cerebral thrombosis	Immune cells phagocytosed the MPs in the bloodstream (MPL-cells) MPL-Cells obstruction in the cerebral vasculature. Decreased blood perfusion levels in the brain Vascular embolism Abnormalities in neurobehavioral regulation Weight loss Cells obstruction was higher by larger MPs size		Not given	Huang et al. [19]
ApoE^−/−^ mice	20 nm and 10 μm	PS	100 mg/kg body weight	Subcutaneous injection	16 weeks	Atherosclerosis	Elevated lipid contents Increased inflammation Higher atherosclerotic plaque formation. These conditions were more severe in MPs treated atherosclerotic groups.		Not given	Zhang et al. [60]
Male ApoE^−/−^ mice (C57BL/6 background)	200 nm	PS	5 and 10 mg/kg body weight	Gavage	3 months	Lipid metabolism and atherosclerosis	Plaque formation in the aortic arch. Decreased blood flow velocity. High lipid and collagen deposition in the aortic root and liver Severe fibrosis. Liver injury. Abnormal lipid levels Increased oxidative stress. The severity in these conditions was concentration dependent.		Not given	Wen et al. [62]
Male C57BL/6 mice	0.5 μm and 5 μm	PS	0.1 μg/mL 1 μg/mL	In drinking water	12 weeks	Adiposity and cardiometabolic disease	Body weight increased Increase in body fat Increased adipogenesis Increased glucose and insulin levels and higher homeostatic model assessment of insulin resistance (HOMA-IR) scores Changes in perivascular adipose tissue gene expression Changes in the gut microbiome 0.5 μm with 1 μg/ml have higher effects		Commercial polystyrene beads were used Difficult to accurately model natural MP exposures Did not explore if PS consumption alters gene expression in other fat depots.	Zhao et al. [59]
Male C57 BL/6J mice	20 nm and 100 nm	PS	0.05 or 0.5 mg/kg	Gavage	14 days	Lipid metabolism	Upregulation of glycerophospholipid molecules (phosphatidylserine and phosphatidylinositol) Upregulation of sphingolipids (ceramide and cholesterol derivatives) Disruption of lipid homeostasis Both types of PS-NPs showed similar effects	Autophagic lipolysis and PI3K signaling pathways	Not given	Luo et al. [63]
ApoE^−/−^ mice (C57BL/6 background)	5 μm	PS	1 μg/mL and 10 μg/mL	In drinking water	12 weeks	Atherosclerosis	Weight gain/obesity Abnormal plasma levels of lipid contents High lipid deposition Larger atherosclerotic lesions Suppressed antioxidant enzymes Enhanced endothelial-to-mesenchymal transition Upregulation of bone morphogenetic protein.	BMP pathway and driving EndMT via oxidative stress	Used atherosclerosis-susceptible mice to model high-risk population responses to MPs exposure Current experimental dosing regimens may not represent actual environmental exposure scenarios Causal relationship between elevated ROS and the activation of the BMP pathway were not explored Did not investigate pharmaceutical interventions to ameliorate the exacerbation of atherosclerosis by PS-MPs	Yang et al. [61]

Collectively, observational human studies and mechanistic evidence from in vitro and animal models support a potential link between MNP exposure and adverse cardiovascular effects, including oxidative stress, endothelial dysfunction, inflammation, fibrosis, and thrombosis. However, causal relationships and the translational relevance of preclinical findings to human cardiovascular disease remain to be fully established.

### MNPs and Hypertension

Hypertension is a major, modifiable risk factor associated with increased morbidity and mortality for CVD [48]. Experimental evidence suggests that endothelial injury, platelet activation, inflammation, and MNP accumulation within vascular tissues may contribute to blood pressure dysregulation [49].

A higher concentration of total MPs was observed in the fecal samples of hypertensive patients than in those of healthy controls. PA, polyurethane, chlorinated PE, PVC, acrylates, PP, and fluororubber were the most prevalent MPs. The MPs, ranging in size from 0 to 30 μm, were the most abundant, with a higher distribution in hypertensive patients than in healthy controls, suggesting a potential association between exposure to MPs and the development of hypertension [50].

In addition to observational human studies, several animal studies have examined the potential mechanistic link between MNP exposure and hypertension. In an in vivo study, exposing mice to 1 and 2 mg/L of PS-MPs (5 μm) for 8 weeks significantly increased systolic, diastolic, and mean blood pressure compared with the control group, in a dose-dependent manner. MP-treated mice exhibited dysbiosis of the gut microbiota, disruption of intestinal barrier integrity, increased permeability of metabolic toxins into the systemic circulation, and vascular remodeling, cardiac hypertrophy, and fibrosis, all of which are associated with hypertension [50]. Exposing 5-week-old SD rats to 0.01 mg/g bw/d of 0.3 μm PS-MPs, amine-modified PS-MPs, and carboxylated PS-MPs for 42 days resulted in an increase of mean arterial blood pressure by 22–40%. Modified MPs exerted more adverse effects than unmodified MPs; amine-modified PS-MPs significantly increased blood pressure and myocardial hypertrophy. Proteomic analysis of differentially expressed proteins in the heart suggested activation of coagulation factor FXII, negative regulation of proteolysis, and, collectively, downregulation of kininogen. MP exposure was associated with ERK activation, the downregulation of bradykinin, and inhibition of the downstream nitric oxide signaling pathway [51].

Overall, current evidence linking MNP exposure to hypertension remains largely derived from experimental animal models, which support a potential role for MNP-induced vascular inflammation, endothelial dysfunction, and gut–vascular axis alterations in blood pressure regulation. However, human data are still limited to observational associations.

### MNP-Induced Gut Dysbiosis: Implications for Cardiovascular Disease

Increasing experimental evidence suggests that MNP exposure may influence cardiovascular health by modulating gut microbiota. In mice, exposure to PS-MPs leads to depletion of canonical short-chain fatty acid (SCFA) producers and an increase in trimethylamine N-oxide (TMAO) levels, changes commonly associated with the development of hypertension. In this murine model, both a high-fiber diet and broad-spectrum antibiotics partially counteract these effects by restoring eubiosis and blood pressure [50]. Similar gut–heart alterations, including increased TMAO and gut permeability, have been implicated in experimental heart-failure models [52]. In mice, inhalation exposure to 80 nm PS-NPs was associated with reduced cecal propionate levels, impaired left ventricular contractility, and myocardial fibrosis. Transcriptomic analyses suggested downregulation of cardiac fructose-1,6-bisphosphatase-1 (FBP1); interventions such as propionate replenishment or pharmacological activation of FBP1 restore cardiac function and blunt fibrotic signaling [53].

SCFAs are thought to exert cardioprotective effects through anti-inflammatory signaling and maintenance of epithelial barrier integrity [54]. Beyond SCFAs and TMAO, additional microbiota-derived mediators, including bile acids and gut neuroendocrine signaling molecules, may also contribute to cardiovascular regulation [55].

MNP-induced dysbiosis may further contribute to cardiovascular dysfunction by increasing intestinal permeability and the translocation of bacterial extracellular vesicles, which can activate endothelial and inflammatory pathways [56]. Altered microbiome profiles have also been associated with vascular dysfunction and impaired cardiac performance in clinical studies [57]. Collectively, these findings support a possible mechanistic link between MNP-associated gut dysbiosis, systemic inflammation, and cardiovascular remodeling. However, most currently available mechanistic evidence derives from animal and experimental studies, while longitudinal human studies quantifying the impact of chronic exposure to distinct MNP fractions (e.g., PET, PE, PVC) remain missing. Determining if each polymer drives unique dysbiosis signatures and specific cardiovascular risks is an urgent priority. Current estimates indicate that daily dietary intakes from drinking water, seafood, and table salt range from micrograms to low milligrams per person, highlighting the translational relevance of this emerging threat.

### MNP and Metabolic Dysregulation: Effects on Body Weight and Lipid Metabolism

**Fig. 3:**
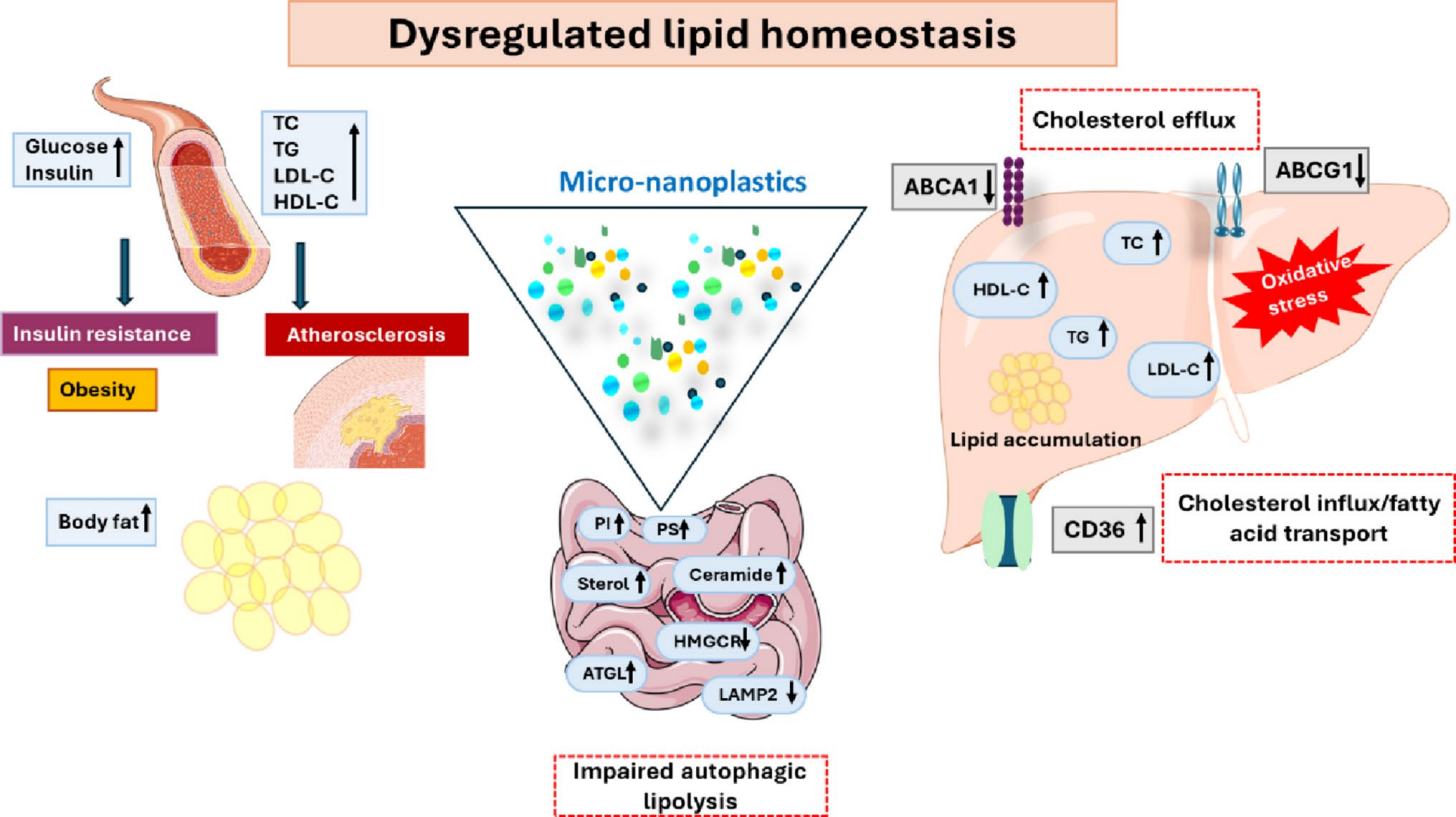
Illustration of micro-nanoplastics in disrupting lipid metabolism. Micro-nanoplastics (MNPs) increase body fat, enhancing circulating levels of glucose and insulin, causing higher insulin resistance. MNP induced elevated levels of circulating and hepatic TC, TG, LDL-C, and HDL-C, and oxidative stress-associated impairment of cholesterol transport and efflux is linked with the progression of atherosclerosis. MNPs alter lipid homeostasis and disrupt the lipid-related signaling pathway in the intestines. *TC* total cholestrol, *TG* triglyceride, *LDL-C* low-density lipoprotein cholesterol, *HDL-C* high-density lipoprotein cholesterol, *PS* phosphatidylserine, *PI* phosphatidylinositol, *LAMP2* lower lysosomal-associated membrane protein 2, *ATGL* adipose triglyceride lipase, *HMGCR* 3-hydroxy-3-methylglutaryl-CoA reductase. Figure created using resources from Servier Medical Art (https://smart.servier.com), licensed under CC BY 4.0 (https://creativecommons.org/licenses/by/4.0/) and Bioicons (https://bioicons.com)

Preclinical studies suggest a role of MNPs in the pathogenesis of metabolic and CVD, such as diabetes, obesity, and atherosclerosis, by disrupting lipid homeostasis [40, 58]. Experimental studies in mouse models provide evidence that exposure to MNPs affects body weight, intestinal barrier injury, alters systemic lipid metabolism, leading to lipid accumulation in vascular and metabolic tissues, and enhances the progression of atherosclerotic plaques (Fig. 3). Mice injected with amine-modified PS-NPs (80 nm) and carboxylated PS-NPs at 5 mg/kg showed a significant reduction in body weight, suggesting a negative NP impact on mice’s health [46]. On the other hand, Zhao et al. [59] showed a significant increase in body weight and a higher percentage of body fat in mice exposed to 5 μm and 0.5 μm PS bead. These apparently divergent findings suggest that the metabolic effects of MNPs may depend on particle size, surface modification, exposure route, dose, and experimental model. PS beads (0.5 μm) at a 1 μg/ml dose demonstrated a significant increase in plasma HDL, higher plasma glucose and insulin levels, and higher insulin resistance (HOMA-IR) scores, suggesting the potential role of MNP exposure in promoting obesity and metabolic dysfunction. Furthermore, Zhang et al. [60] reported a significant exacerbation of plaque in atherosclerotic mice after subcutaneous injection of PS-MNPs (20 nm and 10 μm; 100 mg/Kg) compared with untreated atherosclerotic mice. However, MNP treatment did not result in significant changes in body weight in atherosclerotic mice. In ApoE^−/−^ mice, MNP exposure was associated with reduced CD31 expression, increased lipid deposition in the aortic atherosclerotic plaque and significant alterations in serum lipid profile, including increased total cholesterol (TC), triglycerides (TG), low-density lipoprotein cholesterol (LDL-C), and high-density lipoprotein cholesterol (HDL-C). These findings suggest disruption of systemic lipid metabolism following MNP exposure. Additionally, Yang et al. [61] reported an association between significantly elevated plasma TG, TC, and LDL levels and the atherosclerosis progression in PS-MP-exposed ApoE^−/−^ mice. PS-MPs induced oxidative stress in aortic tissue by significantly suppressing the activities of antioxidant enzymes (Glutathione and total superoxide dismutase) and increasing the activity of lipid peroxidation marker (Malonaldehyde). The significant increase in the expression of Vimentin, Twist1, and Snail1 in endothelial cells of PS-MPs-treated mice indicates enhanced endothelial-to-mesenchymal transition (EndMT) with a subsequent increase in transformed mesenchymal cells. Significant increases in mRNA expression of BMP2, BMP4, BMP9, ALK2, and ALK3 in MPs indicate the involvement of bone morphogenetic proteins (BMPs). These findings support the hypothesis that PS-MPs may induce atherosclerosis and cardiovascular toxicity by activating the BMP pathway and driving EndMT via oxidative stress [61].

In addition to lipid deposition in atherosclerotic plaque in the aortic root and serum lipid levels, Wen et al. [62] reported a significant increase in TC, TG, LDL-C, and HDL-C in the livers of 200 nm PS-NP-exposed ApoE^−/−^ mice in a concentration-dependent manner. NPs-exposed mice documented down-regulation of cholesterol transport genes, ABCA1 and ABCG1, and upregulation of fatty acid transport gene CD36 in the liver and increased hepatic oxidative stress, underscoring the oxidative stress-associated impairment of cholesterol transport and efflux. These alterations may contribute to atherosclerosis development.

At the intestinal level, Luo et al. [63] showed that ingestion of PS-NPs by male C57 BL/6 J mice altered the lipid profile. Glycerophospholipid (GP) molecules, such as phosphatidylserine (PS) and phosphatidylinositol (PI), and sphingolipids (SP), including ceramide (Cer), as well as sterol lipids like cholesterol derivatives, were upregulated. Furthermore, NPs affected the autophagic lipolysis pathway. This was evidenced by the presence of autophagosomes, co-localization of typical autophagic lipolysis proteins in intestinal sections, lower lysosomal-associated membrane protein 2 (LAMP2) levels, and elevated adipose triglyceride lipase (ATGL) levels in intestinal homogenates. Additionally, a significant decrease in 3-hydroxy-3-methylglutaryl-CoA reductase (HMGCR) suggested an alteration in the PI3K-signaling pathway. These findings suggest that NPs altered lipid homeostasis and disrupted the lipid-related signaling pathway in mouse intestines. Collectively, these predominantly preclinical studies support a potential association between MNP exposure and multiorgan disturbances in lipid homeostasis, including intestinal and hepatic metabolic dysregulation and vascular lipid accumulation. These alterations may contribute to a cardiometabolic phenotype associated with increased cardiovascular risk. However, the translational relevance of these findings to human disease remains to be fully established.

### MNP-Associated Thrombotic and Hemorrhagic Complications

Emerging evidence suggests that MNP exposure may interfere with hemostatic balance and contribute to coagulation abnormalities potentially associated with thrombotic and hemorrhagic complications [40, 64]. Experimental studies indicate that particle size, surface modification, and exposure dose may significantly influence coagulation responses and thrombus formation [31, 65]. Sivan et al. [66] found elevated MP levels in CVD patients compared to controls. Higher concentrations of D-dimer and fibrinogen were positively associated with MPs levels. These findings support a possible association between increased MP burden, vascular inflammation, and a pro-thrombotic profile in CVD patients. In vivo studies showed that exposing chickens to PS-MPs (5 μm) led to cerebral hemorrhage, microthrombus formation, and loss of Purkinje cells. In detail, intracerebral hemorrhage increased infiltration of inflammatory cells and activated the ASC-NLRP3-GSDMD signaling pathway, suggesting activation of pyroptosis-related pathways. PS-MPs exposure also disrupted mitochondrial dynamics, leading to mitochondrial dysfunction and activation of AMPK signaling [64].

In murine models, circulating immune cells were shown to phagocytose PS-MPs. These immune cells, called MPL-cells, caused obstructions in the capillaries of the brain cortex. Such blockages, similar to thrombus development, decrease blood flow and impair neurological function in mice. MPL-cell obstruction in the cerebral vasculature of mice treated with 2 μm and 0.08 μm PS-MPs was significantly lower than that of 5 μm. The obstructed cells were cleared more readily in smaller MP groups (5 μm) than in larger groups. This suggests that MP size is a key factor in shaping MPL-cell obstruction and supports the hypothesis that MPs may regulate cell obstruction and interfere with local blood circulation, rather than directly penetrating tissue. Immune factors released by MPL-cells may induce tissue factors, potentially contributing to platelet activation, proteolytic activation of the coagulation cascade, and thrombin generation. Moreover, the MP presence in the bloodstream may induce obstruction in areas where sedimentation occurs in the lining of blood vessels. The potential clinical relevance of these mechanisms may be greater in individuals with pre-existing thrombotic or cardiovascular conditions, such as cerebrovascular infarction and MI [19]. However, their translational significance in humans remains to be established. Overall, current evidence linking MNP exposure to thrombotic and hemorrhagic complications derives predominantly from experimental animal models and mechanistic studies, whereas human data remain limited to observational associations and biomarker correlations.

### Mechanisms of MNP-Induced Cardiovascular Disease

#### Internalization by Blood Cells

**Fig. 4:**
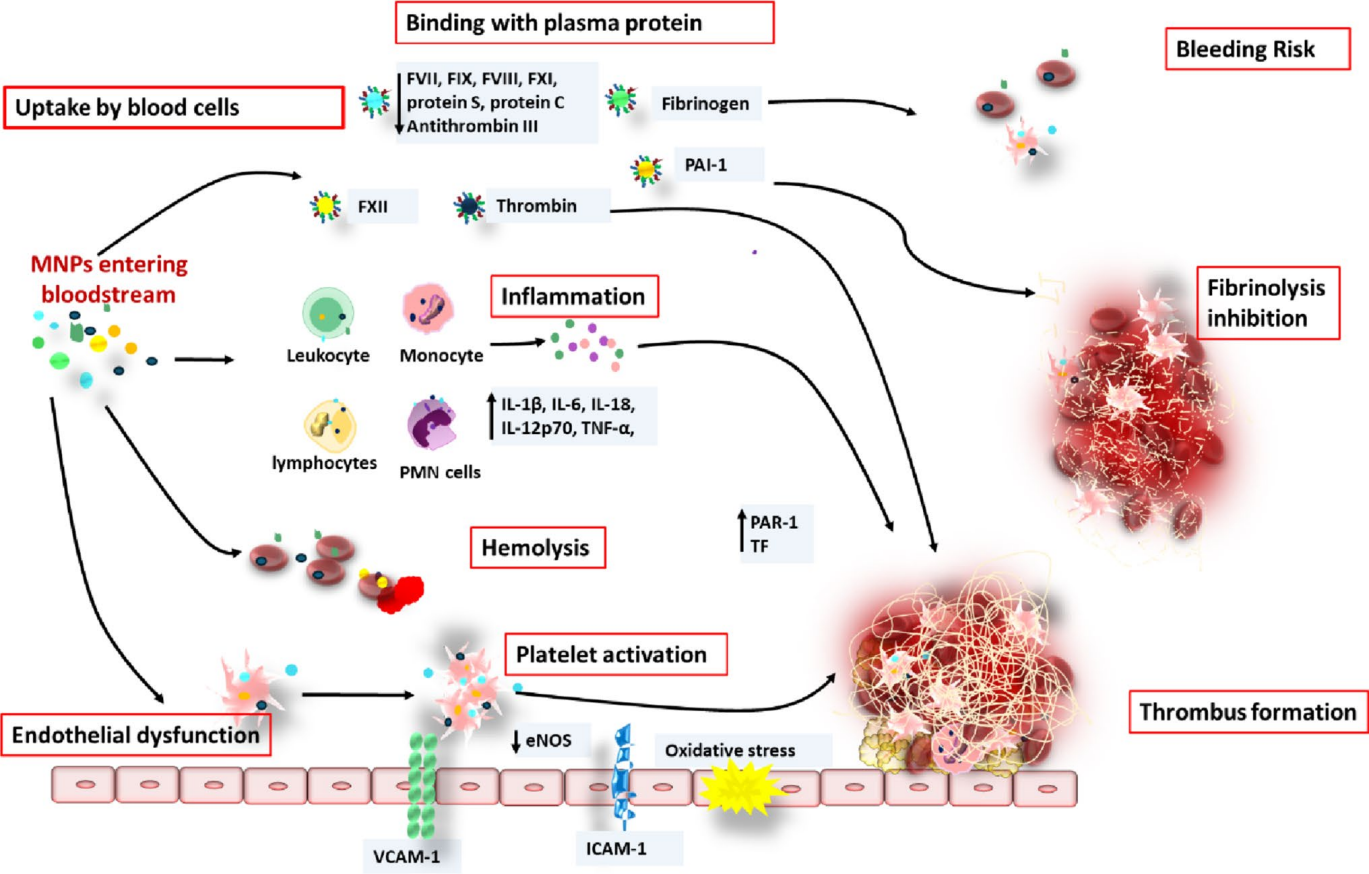
Schematic illustration of micro-nanoplastics (MNPs) effect on inflammation and hemostasis. MNPs are associated with disrupting the balance between pro-coagulation and anticoagulation mechanisms in the coagulation system, depending on the type of protein sorption and the physicochemical characteristics of MNPs. MNPs induce a chronic inflammatory state and oxidative stress that can lead to persistent endothelial dysfunction and platelet activation, resulting in a higher risk of thrombus formation. Binding of MNPs with coagulation factors could deplete them in plasma, resulting in decreased thrombin formation and may lead to bleeding risk. *PMN* polymorphonucleated cells, *IL* interleukin, *PAI-1* plasminogen activator inhibitor-1, *PAR-1* protease-activated receptors-1, *TF* tissue factor, *eNOs* endothelial nitric oxide synthase, *ICAM-1* intracellular adhesion molecule-1, *VCAM-1* vascular cell adhesion molecule

Experimental and biomonitoring studies suggest that plastic particles can enter the human bloodstream following environmental exposure [67]. NPs, by entering the bloodstream, can directly or indirectly interact with different blood cell types such as leukocytes, erythrocytes, and platelets or blood components including various complement proteins and immunoglobulins [32] (Fig. 4). These interactions may influence cellular structure and function. Amine-modified PS-NPs (100 nm) showed dose-dependent uptake and adhesion to RBCs, accompanied by alterations in erythrocyte morphology and aggregation [35].

Complementing these findings, Arribas Arranz et al. [32] showed that exposure of different NPs (PS, amine-modified PS, carboxylated PS, PET, and PLA) to peripheral blood samples from medium-aged healthy donors interacted with all blood cell subtypes at a similar rate. Monocytes exhibited the highest internalization rate, which is consistent with their role in the phagocytic system. The carboxylated PS-NPs displayed significantly higher uptake capacity than amine-modified PS-NPs and pristine PS-NPs in the three cell populations (monocytes, polymorphonuclear cells, and lymphocytes). Polylactic acid NPs (PLA-NPs) exhibited higher uptake in the three white blood cell populations, with significant differences between polymorphonuclear cells and monocytes. On the other hand, PET-NPs uptake showed only a significant difference in monocytes compared to pristine PS-NPs, suggesting that the internalization pattern differs depending on NP nature and surface modification. A recent study exposed human blood to palladium-doped PS-NPs and detected their presence in 37 primary immune-cell subpopulations using single-cell mass cytometry, demonstrating widespread NP uptake across immune-cell populations. Similar findings were observed in mice, where the NPs accumulated in immune cells, including monocytes, macrophages, and dendritic cells, within the liver, blood, and spleen [68]. Collectively, these experimental studies indicate that NP uptake by circulating blood and immune cells is strongly influenced by particle size, composition, and surface chemistry, potentially contributing to systemic inflammatory and thromboinflammatory responses.

#### Blood Cells Count

Experimental and observational studies suggest that MNP exposure may induce hematological alterations and affect circulating blood cell populations [69]. A significant positive correlation was observed between high MP levels and elevated numbers of natural killer cells (CD3-/CD56 + CD16+ cells) and B cells (CD3-/CD19 + cells) in ACS patients, suggesting that MPs might promote atherosclerosis progression through immune cell activation. Conversely, no significant correlations were observed between MPs and monocytes or T cells. However, the correlation of MP content with specific lymphocyte subpopulations varied by type: PE, PVC, and PP showed positive correlations with both B cells and NK cells, whereas PS showed a negative correlation with T suppressor cells (CD3+/CD8 + cells) [43]. Mice exposed to PS, amine-modified PS, and carboxylated PS-NPs indicated a significant increase in white blood cell count, consistent with a systemic inflammatory response. Amine-modified PS-NP exposure was also associated with increased platelet counts, although the difference did not reach statistical significance. There was no significant difference in mean platelet volume, granulocyte, erythrocyte, and hemoglobin levels in different groups. However, mice exposed to PS (20 mg/kg) and carboxylated PS-NPs (0.5 mg/kg) indicated a significant increase in hemoglobin levels [46]. On the other hand, Zhao et al. [59] showed no changes in blood cell counts for most cell types between the control and 5 μm and 0.5 μm PS bead-exposed groups of mice. However, there was an increase in levels of eosinophils, basophils, and monocytes in the 5 μm PS group. These differential effects may reflect variations in particle size-dependent biodistribution and tissue accumulation. Overall, MNP exposure may affect blood cell populations and hematological parameters, although current evidence remains largely based on experimental studies.

#### Inflammation

Experimental and clinical evidence suggests that NPs may promote inflammatory responses potentially involved in cardiovascular disease progression, particularly atherosclerosis [70] (Fig. 4). Positive correlation between the levels of proinflammatory cytokines (IL-1β, IL-6, IL-18, and TNF-α) and MNPs concentrations in the coronary blood of MI patients suggests their pro-inflammatory properties. Of note, correlation differs with MNP type, PS levels were positively correlated with both IL-1β and IL-6; however, PVC levels showed a positive correlation with IL-1β, IL-6, IL-18, and TNF-α. At the same time, PE and PA66 levels showed no positive correlation with these inflammatory cytokines. In coronary thrombi, PVC levels were positively associated with elevated pro-inflammatory cytokine levels and the expression of both CD3 (lymphocyte) and CD68 (macrophage), suggesting a link between PVC burden and inflammatory cell infiltration in coronary thrombi, and a possible contribution of MNPs to inflammatory-cell recruitment within atherosclerotic lesions [22]. Notably, PET-NP stimulation of peripheral monocytes from MI patients exhibited higher mRNA expression levels of IL-1β, IL-18, IL-6, and TNF-α, and lower IL-2 expression [1]. In ACS patients, MP content showed a significantly positive correlation with IL-6 and IL-12p70 levels, suggesting their potential role in coronary pathologies. PVC showed a positive correlation with IL-6, and PE and PS were positively correlated with IL-12p70. PA66 showed anticorrelation with IL-1β and IL-17. PE was negatively correlated with IFN-γ, whereas PS was positively correlated with IFN-γ. The heterogeneous effects of MPs on inflammatory cytokine production could be due to variation in the physicochemical properties of different MP materials [43]. PS-MPs increased the levels of inflammatory markers, including IL-1, IL-1β, and intracellular adhesion molecule-1 (ICAM-1) in both myocardial tissue and serum, and decreased the IL-6 expression level in the myocardial tissue of rats. The observed reduction in myocardial IL-6 expression may reflect secondary metabolic alterations induced by PS-MPs exposure [44].

In the mouse model, PS, amine-modified PS, and carboxylated PS-NPs significantly increased serum levels of IL-6 and monocyte chemoattractant protein-1 (MCP-1) in a dose-dependent manner compared to the control group. The increased expression of Janus kinase 1 (JAK1) and signal transducers and activators of transcription-3 (STAT3) in the aortic arch protein provided further evidence of inflammation in the blood vessel [46]. Compared with normal control and untreated atherosclerotic mice, both 20 nm and 10 µm MP-treated atherosclerotic mice showed significantly higher production of TNF-α, IL-6, and IL-1β, consistent with an enhanced inflammatory response [60]. IL-6 and Mcp-1 genes were upregulated in the perivascular adipose tissue sample of 0.5 μm PS-exposed mice [59]. Exposure of Human Coronary Artery Smooth Muscle Cells to virgin” or photo-degraded PE and PS-MPs diminished vascular smooth muscle cells (VSMCs) viability and triggered pathological changes such as altered migration and proliferation. RUNX-2 and galectin-3, which regulate cardiovascular pathology, were upregulated along with caspase-1, an essential component of the inflammasome complex, suggesting potential health MP risk [71]. Overall, current evidence indicates that MNP exposure may modulate inflammatory pathways through cytokine dysregulation, inflammasome activation, and immune-cell recruitment. However, most mechanistic evidence derives from experimental and ex vivo studies, whereas human data remain largely associative.

#### Endothelial Dysfunction

Experimental evidence suggests that vascular endothelium represents a major target of MNP-induced injury [72]. MNP exposure has been associated with endothelial dysfunction through increased oxidative stress and impaired nitric oxide (NO) bioavailability [72–74]. Endothelial dysfunction is a key factor in inducing atherosclerosis and thrombus formation [75]. Incubating porcine coronary artery endothelial cells (PCAECs) and PCA with 25 nm PS-NPs resulted in increased senescence-associated β-galactosidase activity and increased expression of p53, p21, and p16 proteins, leading to inhibition of proliferation in a dose-dependent manner. NPs-induced downregulation of endothelial nitric oxide synthase (eNOS) expression, causing impaired endothelium-dependent vasorelaxation and promoting reactive oxygen species creation in endothelial cells, which was associated with high NADPH oxidase expression, with subsequent downregulation of Sirt1 expression [76]. Notably, PS administration, amine-modified PS, and carboxylated PS-NPs in mice caused structural disintegration in endothelial cells, leading to pyknotic or exfoliative endothelial cells. However, modified PS exhibited greater cell damage than unmodified PS by causing vacuolation of the endoplasmic reticulum and cytoplasmic accumulation, resulting in cell abscission and death [46].

Collectively, these findings suggest that MNP exposure may promote a chronic inflammatory state that leads to persistent endothelial dysfunction, resulting in a higher risk of thrombus formation over time; however, further studies are needed to investigate the MNP presence in human thrombi and cohort studies to compare the effects of their presence or absence in humans [70]. Incubation of human umbilical vein endothelial cells (HUVECs) with PS-MPs < 5 μm triggered endothelial activation, as evidenced by significant upregulation of ICAM-1 and VCAM-1. Spiking PS-MPs into whole blood at reported plasma concentrations and perfusing over collagen-coated surfaces showed that activated endothelium led to significant enhancement of platelet deposition to collagen, with consequent development of larger, denser thrombi that became unstable and embolic at higher PS-MP concentrations. PS-MPs reduced the lag time to fibrin formation and maximal turbidity, suggesting denser clot formation. This study found that MNP presence in the human bloodstream enhances thrombus formation by shifting endothelial phenotypes toward more pro-inflammatory and prothrombotic conditions, supporting a potential role of circulating MNPs in endothelial activation and thrombus formation [77].

Atherosclerosis progression is associated with abnormal expression of adhesion molecules, matrix metalloproteinases (MMP), chemokines, and endothelial protective proteins [78]. Mice exposed to 5 μm PS-MPs exhibited significant upregulation of ICAM1, VCAM1, CCL-2, and MMP9 in a dose-dependent manner [61]. Murine cell lines, including myocardial endothelial cells and monocytic J774A.1 cells exposed to 1 μm carboxylated PS-MPs at different concentrations, exhibited increased expression of adhesion molecules in endothelial cells with subsequent adhesion of leukocytes. Similarly, mice exposed to PS resulted in increased aortic expression of cytokines and adhesion molecules. Moreover, neutrophils were identified as the PS-clearing leukocyte population in blood [79]. Finally, exposure of 80 nm PS, amine-modified PS, and carboxylated PS-NPs to EA.hy 926 endothelial cells resulted in suppression of cell activity, cell membrane damage, oxidative stress, and significantly inhibited cell migration. Amine-modified PS-NPs caused the most serious damage among the three NPs. Kyoto KEGG (Encyclopedia of Genes and Genomes) enrichment analysis of candidate target genes indicated the primarily enriched pathways included endocytosis, viral carcinogenesis, and complement and coagulation cascades after PS-NPs exposure. The amine-modified PS-NP-treated cells showed candidate target genes, including fluid shear stress and atherosclerosis, cell cycle, Notch, TNF, and mitogen-activated protein kinase (MAPK), whereas carboxylated PS-NPs-treated cells revealed the most enriched pathways, including MAPK, cytokine-cytokine receptor interaction, and lipid and atherosclerosis. *CEBPB* (CCAAT Enhancer Binding Protein Beta), a gene within the inflammation-related TNF signaling pathway, was upregulated after NPs exposure and was confirmed to be a target of miR-1908-5p, suggesting that NPs-induced activation of *CEBPB* might enhance inflammatory injury to vascular endothelial cells [80]. Altogether, current evidence from in vitro, ex vivo, and animal studies indicates that MNP exposure may impair endothelial homeostasis through oxidative stress, inflammatory activation, upregulation of adhesion molecules, and pro-thrombotic signaling.

### MNPs and Platelet Function: Clinical and Translational Evidence

Platelet activation plays a crucial role in CVD by triggering inflammatory cascades and thrombus formation [81]. In this scenario, MNPs have been proposed to contribute to thrombosis through endothelial dysfunction, increased platelet deposition, and coagulation imbalance [70] (Fig. 4). Experimental studies suggest that MNPs may promote oxidative stress and inflammation, both recognized activators of platelet function and coagulation pathways. Moreover, MNPs within the vascular lumen provide a basis for clot formation and serve as surfaces that facilitate platelet adhesion, aggregation, and fibrin deposition [72]. Indeed, Wu et al. [24] found a positive association between MNPs load in human arterial thrombi and platelet count, supporting a possible association between MNP burden and thrombotic processes in vivo. However, Arribas Arranz et al. [32] reported no significant effect of several NP types on platelet activation in whole blood after 24 h, underlining how experimental context and particle chemistry can dramatically influence outcomes. Conversely, Burgos et al. [82] examined the effects of acrylamide (AA) and bisphenol A (BPA), toxic chemicals found in plastics, on platelet physiology. While neither AA nor BPA induced aggregation alone, both compounds, when combined with sub-threshold concentrations of agonists (ADP or PMA), significantly enhanced platelet aggregation, mainly through increased reactive oxygen species and protein kinase C (PKC) activation. In sum, these results suggest that plastic-derived chemicals may sensitize platelets to agonists, increasing the risk of thrombosis in exposed individuals.

#### In Vitro Studies

In recent years, a growing number of in vitro studies have highlighted the NP ability to interact directly with blood components, mostly RBCs [35] and platelets [31], thereby influencing hemostatic balance. Notably, the thrombotic NP potential is not determined by a single factor but rather results from a complex interplay of multiple physicochemical parameters, including particle size, surface area, and charge, chemical functionalization, and concentration [38, 83].

Among blood components, platelets appear more sensitive to NPs, with several in vitro models reporting their capacity to modulate platelet activation and aggregation, key events in the pathogenesis of thrombotic disorders [84, 85]. Due to their small size and high surface reactivity, PS-NPs can engage directly with platelet surface receptors, such as GPIIb/IIIa integrins, which mediate fibrinogen binding and facilitate inter-platelet crosslinking during thrombus formation [86]. One of the foundational studies in this field, conducted by Smyth et al. [31], demonstrated that polystyrene latex nanoparticles (PLNPs) with 50 and 100 nm diameters, functionalized with unmodified, aminated, or carboxylated surfaces, induced significant platelet aggregation via activation of the GPIIb/IIIa receptor. Of note, 50 nm aminated PLNPs were able to trigger platelet clustering even in the absence of soluble agonists, likely through a mechanical “bridging” mechanism. This pro-aggregatory effect was completely inhibited by eptifibatide, confirming GPIIb/IIIa as a central mediator [31]. In addition to integrin-mediated pathways, P-selectin (CD62P) has emerged as a key molecular marker and mediator in PS-NPs-induced platelet activation. As reported by Lett et al. [29], aminated PS-NPs significantly increased P-selectin surface expression on human platelets, promoting the establishment of platelet–leukocyte aggregates, a hallmark of thrombo-inflammatory responses. Additionally, these effects were observed in vivo, where amine-functionalized PS-NPs reduced arterial occlusion time on a murine thrombosis model, suggesting that P-selectin upregulation contributes to both platelet activation and thrombus propagation under physiological flow conditions. In support of this receptor-centric mechanism, Zia et al. [83] further showed that polystyrene and platinum nanoparticles of varying sizes (25–201 nm for PS; 7–73 nm for Pt) induced platelet aggregation in a surface area-dependent manner. The aggregation was inhibited by blocking αIIbβ3 integrin, and was dependent on Src and Syk kinase signaling, implicating ITAM-bearing receptors such as GPVI and CLEC-2 in the activation cascade. A current study by Trostchansky & Alarcón [87], demonstrated robust carboxylated PS-NPs induced platelet activation as evidenced by cytoskeletal remodeling, pseudopod formation, and concentration-dependent CD63 externalization, underscoring a similar mechanistic pattern to strong physiological agonists such as thrombin. The non-saturable uptake of PS-NPs by platelets could be attributed to non-specific, high-capacity mechanisms, such as hydrophobic interactions and pseudo-phagocytosis, thus suggesting that the increased accumulation of PS-NPs may increase with higher environmental exposure. Furthermore, PS-NPs enhance coagulation indirectly by integrating into fibrin networks and functioning as catalytic surfaces for the Ca²^+^-dependent assembly of coagulation factor complexes to support the formation of stable thrombi.

Interestingly, PS-NPs can also interact with plasma coagulation factors, potentially altering thrombin generation. Oslakovic et al. [27] demonstrated that aminated PS-NPs of 57 and 330 nm incubated in human plasma were capable of binding coagulation factors VII (FVII) and IX (FIX), key upstream mediators of the extrinsic pathway. Interestingly, this interaction led to a decrease in thrombin generation, suggesting that, under certain conditions, PS-NPs may exert anticoagulant-like effects by sequestering essential coagulation factors and impairing cascade progression. In contrast, carboxyl-modified NPs (negatively charged) of larger size (220 nm) were able to activate the intrinsic coagulation pathway, similarly to kaolin used in APTT assays, whereas smaller particles (24 nm) did not induce this activation. The effect is attributed to the ability of larger particles to support the assembly of multiple protein complexes (including FXII, FXI, HMWK, and kallikrein) on their less curved surfaces. The study emphasizes that the NP physicochemical properties, especially size and surface functional groups, play a critical role in modulating coagulation and proinflammatory pathways. These findings highlight the dual, context-dependent NP role in modulating coagulation, potentially promoting thrombosis through platelet activation while simultaneously dampening thrombin generation via factor binding [27]. Similarly, Sanfins et al. [28] employed a tissue-factor–free thrombin-generation assay to probe the intrinsic coagulation pathway in citrated plasma. They tested carboxylated PS-NPsof different diameters (24–220 nm) and found that only the larger particles (60–220 nm) triggered thrombin generation, implicating contact-activation factors in this process. Moreover, thrombin production increased with particle size: 220 nm carboxylated PS-NPs had the strongest effect, 60 nm particles induced less, and the smallest (24 and 26 nm) failed to start thrombin formation. High-molecular-weight kininogen and factor XII bound to 60, 93, and 220 nm carboxylated PS-NPs but not to 26 nm particles, demonstrating a curvature‐dependent modulation of intrinsic pathway enzymatic activity. Complementing these findings, Sheng et al. [23] demonstrated that 100 nm PS-NPs rapidly adsorb a discrete set of plasma proteins, notably F XII and plasminogen activator inhibitor-1 (PAI-1), forming a “protein corona” that accelerates in vitro thrombus formation. Proteomic profiling and molecular dynamics simulations revealed selective enrichment of intrinsic-pathway factors on the nanoparticle surface, while functional coagulation assays showed enhanced platelet activation and shortened clotting times upon exposure to PS-NPs. The dual effect of FXII enrichment (triggering the intrinsic cascade) together with surface-bound PAI-1 (inhibiting local fibrinolysis) suggests that PS-NPs mediated protein corona formation can synergistically promote both platelet aggregation and fibrin stabilization [23].

In addition, alternative approaches have been used to investigate the effects of NPs on platelet function. Santos-Martinez et al. [88] employed quartz crystal microbalance with dissipation monitoring (QCM-D) to assess platelet microaggregation under flow conditions. Using PS (23 nm) and silica NPs (10–50 nm), their study revealed that even sub-aggregant NP concentrations could induce early-stage platelet clustering, which is undetectable by light transmission aggregometry. This microaggregation was shown to be mediated primarily via thromboxane A₂ (TXA₂) and MMP-2 signaling, as it was inhibited by acetylsalicylic acid and phenanthroline, while ADP receptor blockade had no significant effect. The response was also suppressed by nitric oxide donors and prostacyclin, suggesting that platelets maintained pharmacological sensitivity following NP exposure. Further mechanistic insight into the procoagulant effects of PS-NPs was provided by Christodoulides et al. [38], who investigated whole blood clotting dynamics using thromboelastography (TEG). The authors compared carboxylated, aminated, and unmodified PS-NPs in the 50–500 nm range and demonstrated that carboxylated PS-NPs, mostly in the 50–100 nm range, significantly enhanced coagulation parameters. These included a reduced R-time (clot initiation), increased angle (rate of fibrin formation), and higher maximum amplitude (clot strength). While aminated particles exhibited a similar trend to a lesser extent, unmodified particles had negligible effects. The observed prothrombotic potential was dose-dependent and closely linked to surface chemistry, reinforcing the notion that nanoplastic-induced coagulation is a multifactorial process.

While most studies focus on platelet activation, recent findings have also highlighted the RBC in shaping the procoagulant environment induced by NPs. Kim et al. [35] demonstrated that amine-functionalized PS-NPs induced phosphatidylserine externalization, microvesicle release, and disrupted calcium homeostasis in RBCs, leading to a significant intracellular calcium (Ca²⁺) increase. This calcium influx activated scramblase enzymes, promoting phosphatidylserine externalization on the RBC membrane, a key procoagulant signal. As a result, thrombin generation was markedly enhanced in vitro, and PS-NP exposure accelerated thrombus formation in a rat model. These findings underscore the ability of surface-modified PS-NPs to trigger calcium-dependent prothrombotic pathways, potentially amplifying coagulation not only in platelets but also in RBCs.

Overall, these findings provide converging evidence that NPs can modulate platelet function through receptor engagement, mechanical interaction, and intracellular signaling. Surface charge and particle size emerge as critical variables, with aminated PS-NPs consistently showing the highest pro-aggregatory potential. The NP’s ability to influence both early platelet responses and global coagulation parameters underscores their potential to exacerbate thrombotic risk, mainly in predisposed subjects.

#### In Vivo Studies

Having established NPs driven platelet activation in vitro, subsequent in vivo models have similarly demonstrated a critical NP role in modulating thrombus formation. A foundational experiment conducted by Nemmar et al. [89] on a Syrian hamster model of experimental microvascular thrombosis highlighted the key role of PS-NPs surface functionalization. After intravenous dosing with PS-NPs of 60 nm, unmodified PS-NPs were inert up to 5 mg/kg, carboxylated PS-NPs only inhibited thrombosis at intermediate doses (100–500 µg/kg), whereas amine-modified PS-NPs markedly accelerated thrombus formation at both 50 and 500 µg/kg. Intratracheal instillation of amine PS-NPs (5 mg) likewise increased thrombus size, while unmodified and carboxylated forms remained inactive. Complementary in vitro assays in human platelet-rich plasma showed that aminated PS-NPs both directly induced aggregation and potentiated ADP-driven responses, underscoring a surface-charge–mediated prothrombotic mechanism [89].

Rat ear vein intravital microscopy has further confirmed this charge-dependent thrombogenicity: in Wistar rats, 60–100 nm amine PS-NPs (50–500 µg/kg, IV) significantly shortened the vein occlusion time following laser-induced endothelial injury, whereas unmodified or carboxylated PS-NPs remained inactive. Pharmacological blockade of P-selectin or fibrinogen binding attenuated this effect, implicating platelet adhesion and fibrin deposition as key mediators [90].

More recently, Sheng et al. [23] extended these findings in mice, showing that pre-treatment with PS-NPs significantly shortened tail-bleeding time compared to controls, confirming a systemic procoagulant effect and, in a rat model of venous thrombosis, that intravenous administration of PS-NPs three hours before thromboplastin induction produced a dose-dependent increase in thrombus size and weight. Fluorescence imaging verified PS-NPs accumulation within the clots, and the peak blood concentration of PS-NPs (∼7.8 µg/mL) only modestly exceeded the range of environmentally realistic circulating levels (1.6–7.1 µg/mL) reported [67].

Complementing these hamster and mouse studies, Kim et al. [35] investigated Sprague–Dawley rats pre-treated intravenously with 100 nm amine-modified PS-NPs at 0.25, 0.5, and 1 mg/kg. Three hours post-administration, thrombosis induced by thromboplastin resulted in a pronounced, dose-dependent increase in thrombus size and weight. Mechanistically, the positively charged PS-NPs bind to the phospholipid membranes of platelets and endothelial cells, triggering Ca²⁺ influx, degranulation, and conformational activation of integrin αIIbβ3, thereby driving aggregation. Concurrently, PS-NP surfaces adsorb key coagulation factors (e.g., fibrinogen and factor XII), accelerating thrombin generation and fibrin network formation. Even the lowest dose (0.25 mg/kg) significantly enhanced venous thrombus formation compared to saline controls, confirming that surface-charged PS-NPs act as potent procoagulants in vivo.

Collectively, current evidence from in vitro and animal studies supports a strong influence of NP physicochemical properties on platelet activation and coagulation pathways. Surface charge, particle size, and protein corona formation emerge as critical determinants of thrombogenicity. However, direct clinical evidence linking environmentally relevant NP exposure to thrombotic cardiovascular events in humans remains limited.

### Perspectives, Controversies, and Limitations

Emerging evidence suggests that beyond CVD, MNP-associated platelet activation may also have broader implications in thromboinflammatory conditions, including cancer progression [91–93]. Despite substantial progress in uncovering the prothrombotic effects of MNPs, several controversies and limitations temper the interpretation of current findings. First, the heterogeneity of experimental models, ranging from whole blood and isolated platelets to plasma and animal systems, introduces variability that complicates direct comparisons across studies.

Second, physicochemical differences in particle charge, size, and surface functionalization can produce opposing outcomes, with some formulations promoting coagulation while others exert anticoagulant-like effects by sequestering clotting factors.

Third, most investigations employ high, acute concentrations (≥ 10 µg/mL or ≥ 50 µg/kg) that exceed environmentally realistic exposures (1.6–7.1 µg/mL), making it difficult to assess risk under chronic, low-dose conditions.

Finally, few studies incorporate relevant cofactors such as endothelial cells, leukocytes, or the complement system, potentially overlooking critical interactions that occur in vivo.

Addressing these limitations will require (i) standardized protocols, (ii) systematic dose–response analyses at environmentally relevant levels, and (iii) integrated co-culture or organ-on-chip approaches to more faithfully recapitulate human physiology.

### Therapeutics Target to Mitigate MNP-Induced Cardiovascular Diseases

To date, no specific therapies are available to directly eliminate MNPs from the body, and strategies aimed at enhancing detoxification and excretion pathways remain largely speculative [94]. The strategies targeting MNP-induced inflammation have emerged as potential therapeutic approaches [22]. Antithrombotic drugs primarily target secondary platelet signaling pathways; however, strategies to mitigate the nanoparticle (NP) effects on platelets depend on NP physicochemical properties and may differ from approaches used for conventional platelet-mediated disorders [31]. Antioxidant (N-acetylcysteine), NADPH oxidase inhibitor (apocynin), and Sirt1 activator (resveratrol) treatments reduced NPs-induced senescence and dysfunction in porcine coronary artery endothelial cells. These findings suggest that NPs-induced premature endothelial senescence, at least in part, involves a redox-sensitive eNOS/Sirt1 signaling pathway, which could be a therapeutic target to mitigate NPs-associated CVD [76]. Recently, D’Onofrio et al. [95] provided preclinical mechanistic evidence showing that PCSK9 inhibitor evolocumab mitigated PE and PVC MPs induced redox imbalance, improved mitochondrial metabolism impairment, reduced inflammatory mediators (MCP-1, VCAM1 and ICAM1), activation of autophagic pathway, cell cycle arrest and apoptosis in human aortic endothelial cells (teloHAEC), human umbilical vein endothelial cells (HUVEC), and human coronary artery endothelial cells (HCAEC) through restoring the levels of SIRT6 and Forkhead box O3 (FOXO3A). These findings suggest that PCSK9 inhibition may represent a potential therapeutic strategy to counteract MPs-induced endothelial injury and cardiovascular risk.

Dhakal et al. [96] indicated that significant NPs-induced upregulation of SGLT2 on porcine coronary artery-derived endothelial cells by NPs and Enavogliflozin treatment significantly reduced NPs-induced senescence-associated-*β*-gal activity, cell‐cycle arrest, and senescence markers p53 and p21, suggesting that SGLT2 inhibition prevents NPs-induced endothelial senescence. Additionally, Enavogliflozin lowers oxidative stress by downregulating Nox2 and p22phox. Moreover, SGLT2 inhibition upregulated endothelial nitric oxide synthase expression, leading to improved vascular function.

In sum, these findings highlight that endothelial senescence by NPs, in part, is linked to SGLT2, thereby making it a potential target for preventing environmental pollutant-induced CVD mediated by endothelial senescence and dysfunction. The findings of Zangene et al. [97] showed the cardioprotective effect of resveratrol in PS-MPs-induced cardiotoxicity in a murine model. Resveratrol treatment improved inflammatory cell infiltration, cell arrangement structure, and cardiomyocyte apoptosis, and restored myofiber width and cardiomyocyte area. The levels of fibrosis, inflammation, and apoptosis-related markers, including α-smooth muscle actin, type I and type III collagen, nuclear factor erythroid 2-related factor 2 (Nrf2), TNFα, IL-6, desmin, and caspase-3 [98] were significantly decreased in resveratrol-treated mice. Resveratrol enhanced antioxidant defenses by significantly increasing the levels of catalase, superoxide dismutase, and glutathione, while heat shock proteins (HSPs), including HSP40, HSP25, and HSP70, were significantly reduced. These findings suggest that resveratrol could be a promising therapeutic agent to diminish MNP-induced toxicity. Moreover, Wang et al. [50] assessed the therapeutic potential of a 5% high-fiber diet or antibiotic pretreatment to reduce hypertension in MP-exposed mice by modulating the intestinal flora. Both interventions effectively reduced the MP-induced elevation of systolic, diastolic, and mean blood pressure and decreased vascular media thickness and cardiomyocyte hypertrophy. The high-fiber pretreatment decreased MP-induced left ventricular posterior wall (LVPW), whereas antibiotics showed no significant effects on the interventricular septum (IVS) or LVPW but decreased myocardial interstitial fibrosis.

Overall, these findings suggest that pretreatment with dietary fiber and antibiotics mitigates MPs-induced hypertension and structural damage in mice. The MNP exposure to mice caused myocardial fibrosis by inhibiting Dickkopf-related protein 3 (DKK3) expression. DKK3 overexpression restored autophagic function, reduced cardiomyocyte pyroptosis, and mitigated myocardial fibrosis, supporting a possible protective role of DKK3 signaling against MNP-induced myocardial fibrosis [99]. Overall, currently available therapeutic strategies targeting MNP-induced cardiovascular toxicity remain largely experimental and are predominantly supported by in vitro and animal studies. Further translational and clinical investigations are required to determine whether these approaches may have relevance in humans exposed to environmentally realistic MNP levels.

### MNP Exposure via Medical Devices in Cardiovascular Procedures

Cardiovascular procedures may represent a direct source of short-term intravascular MP exposure via plastic-based intravenous catheters, syringes, or other drug delivery systems [9].

Cardiac invasive catheters are extensively used in clinical cardiology for diagnostic purposes, risk stratification, and interventional guidance. These devices are introduced into heart chambers or coronary vessels to evaluate hemodynamics, reveal coronary obstructions, and perform therapeutic procedures. Invasive hemodynamic measurements obtained via catheterization are essential for diagnosing and managing various forms of heart failure, including heart failure with preserved ejection fraction (HFpEF). In the setting of acute MI, cardiac catheterization is critical for identifying and treating coronary occlusions or unstable lesions. Overall, invasive procedures conducted by catheterization remain the gold standard for hemodynamic evaluation in cardiovascular patients, offering essential clinical insights [100].

Cardiac catheters are typically composed of materials such as stainless steel 316 L, polytetrafluoroethylene (PTFE), and PET, due to their biocompatibility and structural stability post-implantation [101]. In addition, the PTFE and PET are used in vascular grafts for their chemical resistance and mechanical resilience [102]. Moreover, innovative drug delivery strategies are being developed, including the integration of poly (β-amino ester) (pBAE) nanoparticles into polymeric stent coatings to enhance targeted drug release and cellular penetration [103]. Altogether, these factors could affect potential MP release from such catheters, although the clinical relevance of such exposure remains incompletely understood.

Intravenous (IV) infusion systems may contribute to direct intravascular exposure to MNPs during routine clinical care. A study on PVC-based infusion tubes has shown the release of PVC MPs and NPs, ranging from 1003.6 ∼ to 3494.6 particles and 0.042 ∼ to 0.087 μg, respectively, under a stimulating normal infusion scenario. The release of PVC MPs and NPs was also increased with the infusion duration and temperature [104].

Dewika et al. [101] analyzed 21 catheter samples from 7 brands, revealing that significant amounts of MP were released into the human body during cardiovascular procedures. No significant difference in MP counts was found among catheter brands; however, released MPs differed in type, size, and composition, which may result in different biological interactions and exposure profiles. Raman spectroscopy results confirm that the released MP particles were composed of polyurethane, nylon, and PTFE. This supports the idea that both the outer and inner layers of the catheter can undergo mechanical stress and chemical interactions with bodily fluids and drugs administered during procedures, potentially contributing to the leaching of these polymeric materials.

Liu et al. [9] demonstrated that percutaneous coronary intervention (PCI) increases MP levels in patients with coronary artery disease. Laser direct infrared (LDIR) analysis showed significantly higher MP concentration in the post-PCI blood than pre-PCI (93.57 ± 35.95 vs. 4.96 ± 3.40 particles/10mL of blood, *P* < 0.001).

PA, PU, and PET were the major polymers, consistent with the MP types detected in the PCI device washing water. Large MPs were detected in post-PCI (213 μm) than in pre-PCI (50 μm) samples, and an even larger size (336 μm) was detected in device-washing water, supporting the possibility of MP shedding from PCI devices. The MP concentration significantly decreased in post-PCI blood from 0 h to 2 h, and then it remained constant at 4, 8, and 24 h, potentially reflecting MP redistribution or clearance dynamics following the procedure. These findings provide preliminary evidence that PCI devices may contribute to transient intravascular exposure to MP.

A recent observational study by Chen et al. [105] reported MP detection at all catheter tips in 55 intensive care unit (ICU) patients with central venous catheters (CVCs). Different MP types were associated with distinct clinical outcomes in those patients: PE, polyamide, chlorinated PE, and cellulose were associated with an increased risk of thrombosis, while nylon and polylactic acid were associated with central-line-associated bloodstream infections. Nylon, PE, chlorinated PE, polyamide, and cellulose were linked with a high risk of unplanned extubation. Elevated MP levels (> 300 particles per tip) were associated with systemic inflammation, as shown by a 5.8-fold increase in IL-6 levels and showed high diagnostic accuracy for predicting catheter-associated bloodstream infections and unplanned extubation, supporting the hypothesis that catheter-associated MPs may have potential utility as biomarkers of device-related complications, although causal relationships remain to be established. Furthermore, these findings highlight the need for further investigation into safer device materials and standardized exposure assessment strategies.

Collectively, these studies suggest that medical devices may represent an underrecognized source of short-term intravascular MNP exposure. However, current evidence mainly demonstrates particle detection and observational associations, whereas definitive evidence linking device-derived MNP exposure to adverse clinical outcomes remains lacking. Standardized analytical methods, contamination controls, and prospective clinical studies will be essential to clarify the true clinical significance of this emerging issue.

## Conclusion and Future Perspectives

The findings reviewed here suggest that MNPs are biologically active environmental contaminants with the potential to interfere with multiple cardiovascular pathways. Current evidence linking MNP exposure to cardiovascular disease spans different levels of inference, ranging from observational associations in humans to mechanistic and causal evidence derived predominantly from experimental in vitro and animal models. Observational human studies support associations between MNP burden and coagulation abnormalities. The levels of MNP presence in blood, plaque, and coronary thrombi have been associated with inflammatory and coagulation biomarkers. Experimental in vivo and in vitro studies provide biological plausibility for these associations by showing that MNP exposure may promote oxidative stress, endothelial dysfunction, chronic inflammation, intestinal dysbiosis, altered lipid metabolism, and thromboinflammatory responses, potentially contributing to a pro-atherosclerotic and pro-thrombotic environment. MNP interactions with the endothelium, the immune system, and platelets collectively support the hypothesis of an “immuno-thrombotic” axis potentially relevant to cardiovascular risk. At the same time, iatrogenic exposure through medical devices in humans is emerging as an underestimated source of intravascular MNPs, with possible implications for thrombotic and infectious complications in critical contexts. Despite progress, important knowledge gaps remain: the lack of longitudinal human studies on chronic low-dose exposure, systematic comparisons across different polymers, and integrated experimental models that include the endothelium, leukocytes, platelets, and microbiota. Future research should prioritize the development of organ-on-chip models and multicellular co-cultures, as well as targeted preventive and therapeutic strategies (reducing iatrogenic exposure, modulating dysbiosis, antioxidants, SGLT2 inhibitors, targeting redox pathways, and the inflammatory axis).

A further major limitation in our understanding of the cardiovascular effects of MNPs in humans stems from the fact that many mechanisms identified in experimental models have not yet been validated clinically. Most animal studies are short-term, making long-term longitudinal studies necessary.

Further critical aspects to be addressed concern the differential biological behavior of MPs and NPs. Although they are often collectively referred to as MNPs, increasing evidence suggests that particle size critically influences biodistribution and cellular uptake. Specifically, due to their nanoscale dimensions, NPs may more efficiently cross biological barriers, penetrate endothelial and immune cells, interact directly with intracellular organelles, and circulate systemically. This may translate into stronger oxidative, inflammatory, and pro-thrombotic effects than those of larger MPs. On the other hand, MPs may predominantly contribute to local inflammatory responses and act as carriers for adsorbed toxic compounds or microbial components. However, direct comparative studies evaluating the size-specific cardiovascular risk profiles of MPs and NPs under standardized experimental conditions remain lacking. This limitation is further compounded by the greater analytical challenges associated with the detection, characterization, and quantification of NPs in biological matrices, which currently preclude accurate exposure assessment and mechanistic comparisons between MPs and NPs.

Finally, human exposure varies widely by diet, lifestyle, and geographic region, necessitating interdisciplinary and standardized approaches to more accurate risk assessment.

Looking ahead, an interdisciplinary approach integrating environmental toxicology, cardiology, hemostasis, and microbiology will be essential to define the real clinical impact of MNPs and guide health policies and innovations in biomedical materials. Multicenter prospective studies will be required to quantitatively correlate environmentally relevant iatrogenic MNP exposure with clinical outcomes to inform clinical practice and regulatory and device procurement policies.

## Data Availability

No datasets were generated or analysed during the current study.
